# Antimicrobial Activity of *Piper marginatum* Jacq and *Ilex guayusa* Loes on Microorganisms Associated with Periodontal Disease

**DOI:** 10.1155/2018/4147383

**Published:** 2018-09-25

**Authors:** Fredy Gamboa, Camila-Cristina Muñoz, Gloria Numpaque, Luis Gonzalo Sequeda-Castañeda, Sandra Janeth Gutierrez, Nohemi Tellez

**Affiliations:** ^1^Department of Microbiology (School of Sciences) and Dental Research Centre Group (School of Dentistry), Pontificia Universidad Javeriana, Bogotá, Colombia; ^2^Department of Chemistry (School of Sciences), Pontificia Universidad Javeriana, Bogotá, Colombia; ^3^Department of Pharmacy, Faculty of Sciences, Universidad Nacional de Colombia, Bogotá, Colombia; ^4^Dental Research Centre Group (School of Dentistry), Pontificia Universidad Javeriana, Bogotá, Colombia

## Abstract

**Background:**

Chronic periodontitis is a multifactorial infectious disease, where multiple bacteria, such as *Porphyromonas gingivalis*, *Prevotella intermedia*, and *Fusobacterium nucleatum* are implicated. The main purpose of researching natural products is to find substances or compounds with antimicrobial activity.

**Aim:**

The objective of this work was to determine antimicrobial activity from extracts and obtained fractions from *Piper marginatum* Jacq and *Ilex guayusa* Loes on *P. gingivalis* ATCC 33277, *F. nucleatum* ATCC 25586, and *P. intermedia* ATCC 25611.

**Methods:**

Total ethanol extracts were obtained from both plants. Fractions were obtained from total ethanol extracts with amberlite as a stationary phase employing hexane, acetone, and ethanol-water as solvents. Qualitative and quantitative phytochemical characterization was performed on total ethanol extracts from both plants. Antimicrobial activity from total ethanol extracts and fractions from both plants were evaluated on *P. gingivalis* ATCC 33277, *F. nucleatum* ATCC 25586, and *P. intermedia* ATCC by the well diffusion method with Wilkins–Chalgren agar.

**Results:**

*Piper marginatum* Jacq total ethanol extract presented antimicrobial activity against all three bacteria, whereas *Ilex guayusa* Loes was only efficient against *P. gingivalis* ATCC 33277 and *P. intermedia* ATCC 25611, with inhibition halos from 9.3 to 30 mm. *Ilex guayusa* Loes obtained fractions presented antimicrobial activity against all three microorganisms evaluated, with inhibition halos ranging from 9.7 to 18.7 mm. In regards to *Piper marginatum* Jacq fractions, inhibition halos were between 8.3 and 19 mm, against all three microorganisms evaluated; only hexane fraction did not present antimicrobial activity against *F. nucleatum* ATCC 25586.

**Conclusion:**

*Piper marginatum* Jacq and *Ilex guayusa* Loes total ethanol extracts and fractions presented outstanding antimicrobial activity against *P. gingivalis* ATCC 33277, *P. intermedia* ATCC 25611, and *F. nucleatum* ATCC 25586.

## 1. Introduction

Periodontitis is defined as an inflammation that compromises the entire tooth attachment apparatus and is classified as chronic periodontitis, aggressive and associated with systemic diseases [1–5]. Chronic periodontitis is the most frequent form of periodontal disease, resulting in loss of progressive and bone insertion. Additionally, it is characterized by pocket formation that can lead to tooth loss in adults [1, 2, 6]. Inherent host factors, smoking, and environmental factors are important and determinant in its evolution and severity [1, 2, 6, 7].

Chronic periodontitis is a multifactorial infectious disease, where diverse microorganisms are implicated in its etiology, mainly anaerobic Gram-negative bacteria localized in the subgingival biofilm. Due to their virulence and role in periodontal disease development, the following microorganisms stand out: *Porphyromonas gingivalis*, *Prevotella intermedia*, and *Fusobacterium nucleatum* [1, 2, 6–9]. These bacteria can act individually or collectively with other microorganisms and trigger the characteristic inflammation process of periodontal disease [10–13].

Recognizing the importance of these microorganisms in triggering and progress of chronic periodontitis leads to the design of targeted measures, devised to diminish or eliminate these microorganisms from the oral cavity, taking particularly into account oral health impact on general health and quality of life of the patient [5, 7–9].

In different parts of the world, plant species have been used as a source of traditional medicine and alternatively transformed into infectious diseases therapeutic agents [14, 15]. In oral health, many substances obtained from diverse families of plants have presented antimicrobial activity on important oral infections microorganisms [16–18]. Katsura et al. [17] reported bactericidal activity of bakuchiol (obtained from *Psoralea corylifolia*) on different *Streptococcus*, *Enterococcus*, and *Lactobacillus* species and *Actinomyces viscosus* and *P. gingivalis.* Baicalein flavonoid, obtained from *Scutellaria baicalensis* Georgi, and Hydroxychavicol, phenolic compound attained from *Piper betle*, presented antimicrobial activity on *P. gingivalis*, *F. nucleatum*, and *P. intermedia*, relevant microorganisms in periodontal disease [19, 20]. Likewise, other studies demonstrate plant extract effect on antimicrobial activity on periodontal disease microorganisms [21–26].

*Piper marginatum* Jacq is a plant belonging to the Piperaceae family, found in the Caribbean from Guatemala to Brazil. In Colombia, this plant is popularly known as “tooth-healer” and “small cord” [27, 28]. Extracts obtained from *P. marginatum* Jacq leaves offer medicinal benefits, such as antimicrobial, antimycotic, and antiviral for human, animal, and plant diseases [27, 28]. *Ilex guayusa* Loes is a plant from the Aquifoliaceae, and it is found in tropical and subtropical regions. Originated from the Amazon, it is therefore found in Colombia, Ecuador, Peru, Bolivia, and Brazil [29]. This plant is generally known as “guayusa,” a tree that can reach approximately 10 m height. Consumption of leaf infusion produces nervous and muscular stimulation. It has been reported useful in cold treatments, as well as respiratory and digestive illnesses [29].

Due to multiple medicinal possibilities described for these plants, the objective of this study was to determine antimicrobial activity from extracts and fractions obtained from *Piper marginatum* Jacq and *Ilex guayusa* Loes on periodontal disease-recognized microorganisms.

## 2. Materials and Methods

### 2.1. Plant Material Collection and Processing

*Piper marginatum* Jacq and *Ilex guayusa* Loes leaves and inflorescences were collected in the rural zone of Brazil in the municipality of Viotá, Colombia, located on the southwest of the Department of Cundinamarca, under the geographical coordinates 4°27′00″ North latitude and 74°32′00″ West longitude, at an altitude of 567 m above sea level, with a mean temperature of 25°C. From each plant, 1 kg from its shoot system (leaves and flowers) was collected. Fresh material was left to dry at room temperature (RT), with even thin layer distribution of the plant material. Drying racks were located at a pertinent distance from the floor. To avoid contamination by fungi and/or bacteria, plant material was frequently turned to allow air flow for uniform drying and guarantee low humidity content in the samples. Samples from both plants were sent to Universidad Nacional de Colombia and Universidad Javeriana herbariums for taxonomic determination. *Piper marginatum* Jacq was identified with voucher number 575454 and *Ilex guayusa* Loes with voucher number 527191.

### 2.2. Extraction Preparation

For each plant material (*Piper marginatum* Jacq and *Ilex guayusa* Loes), 80 g were individually macerated with 300 mL ethanol and allowed to soak for 2 h in a laboratory bottle. Additional 500 ml ethanol was added to achieve a final volume of 800 mL. The solution was agitated and remained in the dark for 12 h at RT. Subsequently, the solution was filtered to remove saturated ethanol (65 g/m^2^ filter paper grade 3 hw, Munktell, Sweden), and new ethanol was added. This procedure was carried out during 48 h, with solvent change every 12 h. Obtained extracts were concentrated in a rotary evaporator to prepare solutions at 1, 2, and 4 mg/mL with dimethyl sulfoxide (DMSO) for performing biological activity assays.

### 2.3. Total Ethanol Extract: Phytochemical Study

To perform the first phase of the phytochemical run for each plant, 1 g total ethanol extract was macerated with 50 mL ethanol. For each ethanol extract, presence or absence of sesquiterpenic lactones, coumarins, and cardiotonics were determined by ferric hydroxamate, Ehrlich method, fluorescence test, Baljet's test, and Molisch test. For the second phase of the phytochemical run, 25 mL ethanol extract from the first run was mixed with 25 mL petroleum ether. The solution was placed on phase separation funnel to obtain ether and ethanol extracts, respectively. To the ether extract, presence or absence of steroids (Liebermann–Burchard test) and carotenoids (Salkowski technique) was determined. To the ethanol extract flavonoids (Shinoda test, Rosenheim test, and leucoanthocyanidin test), tannins (ferric chloride test) and saponins (froth assay and Rosenthaler test) were assessed. For the third phase of the phytochemical run, 3 mL ethanol extract from the first run was submitted to acid-base extraction with concentrated hydrochloric acid to evaluate alkaloid content through Dragendorff, Valser, Mayer, and Wagner tests.

### 2.4. Total Phenol and Flavonoid Quantification

Phenol and flavonoid quantification was performed from total ethanol extracts for each plant. Total phenol quantification was carried out with the Folin–Ciocalteu test, which relies on phenol reaction with oxidizing agents [30]. To this end, a gallic acid standard curve was prepared ranging from 50 to 500 ppm, with 50 ppm increase. *Piper marginatum* Jacq and *Ilex guayusa* Loes ethanol extracts were diluted at 1,000 ppm with deionized water for total phenol determination. For flavonoid quantification, aluminum chloride was used [31]. For this assay, aluminum chloride dissolved in ethanol reacts with flavonoids present in the sample, producing a yellow complex. A standard curve with quercetin (Sigma-Aldrich) at 1, 3, 5, 7, 9, 11, 13, 15, 18, and 21 ppm was used. *Piper marginatum* Jacq and *Ilex guayusa* Loes ethanol extracts were diluted at 1,000 ppm with deionized water.

### 2.5. Obtaining Fractions

Soxhlet technique was used to obtain fractions using amberlite as solid-phase and three different solvents with distinct polarities from highest to lowest (ethanol : water, acetone, and hexane). To this end, 1 g *Piper marginatum* Jacq and *Ilex guayusa* Loes ethanol extract was used and macerated independently in mortar with 10 g amberlite, until a homogenous mixture was obtained. Following this, the mix was placed on a paper filter to form a thimble placed in the apparatus to be immediately fractioned with solvents in this strict order: ethanol : water (3 : 1 proportion), acetone, and hexane. Solvents were changed every 12 h until three days were completed. After fractions were obtained, they were concentrated in water bath and vacuum chamber, and solutions with DMSO at 2 and 4 mg/ml were prepared to determine the biological activity.

### 2.6. Antimicrobial Activity Evaluation

Antimicrobial activity was evaluated on *P. gingivalis* ATCC 33277, *F. nucleatum* ATCC 25586, and *P. intermedia* ATCC 25611 strains, which were in lyophilized state. Microorganisms were revived in 5 mL thioglycollate broth (BBL™ Fluid, Becton Dickinson and Company) and incubated for eight days at 35°C under anaerobic conditions (Anaerogen, Oxoid). Subsequently, they were seeded on blood agar and Wilkins–Chalgren agar and further incubated for eight days at 35°C under anaerobic conditions (Anaerogen, Oxoid). To verify strain morphology and purity, Gram stains were performed on isolated colonies. To pre-enriched bacteria, three to five colonies from each strain were cultured in 4 mL BHI broth with 5 *µ*g/ml hemin and 1 *µ*g/ml menadione, incubated for 72 h at 35°C under anaerobic conditions (Anaerogen, Oxoid). Finally, from this pre-enrichment, a 0.5 turbid suspension in the McFarland scale was prepared (approximately 1.5 × 10^8^ CFU/ml) [15]. For antibacterial activity, 200 *µ*L bacteria suspension at 0.5 McFarland scale was added to 20 mL Wilkins–Chalgren agar in liquid state and plated into Petri dishes. After solidification under anaerobic conditions, 4 mm wells were made with sterile Pasteur pipettes, and 30 *µ*L total extracts and fractions at 1, 2, and 4 mg/mL were placed and incubated between seven to nine days at 35°C (Anaerogen, Oxoid). As positive controls, erythromycin and ampicillin at 50 IU/ml and 100 *µ*g/mL were used, respectively. DMSO, ethanol, ethanol-water, acetone, and hexane were used as negative controls. After incubation, readings for each test were performed in triplicate by measuring inhibition halos in mm generated by antimicrobial activities in evaluated samples.

## 3. Results

### 3.1. *P. marginatum* Jacq and *Ilex guayusa* Loes Total Ethanol Extract Phytochemical Study

*Piper marginatun* Jacq plant material (80.099 g) submitted to ethanol extract produced 7.3 g total extract, representing a 9.1% yield. On the other hand, 80.834 g *Ilex guayusa* Loes gave a higher yield (12.7%), obtaining 10.3 g. *P. marginatum* Jacq and *Ilex guayusa* Loes, and total ethanol extract secondary metabolites are illustrated in Table 1. Extracts for both plants presented alkaloids, cardiotonics, carotenoids, flavonoids (Shinoda method), and tannins. Additionally, coumarin was only present in the *Ilex guayusa* Loes ethanol extract. Steroids, flavonoids (Rosenheim test and leucoanthocyanidin test), sesquiterpenic lactones, and saponins were not identified in any plant extract. Phenol quantification obtained per gram of total *Piper marginatum* Jacq and *Ilex guayusa* Loes extract was 45.4 and 57.7 mg gallic acid, respectively. Detected flavonoids per gram of *Piper marginatum* Jacq and *Ilex guayusa* Loes total ethanol extract was 0.65 mg and 1.71 quercetin, respectively.

### 3.2. Total Ethanol Extract and Fraction Antimicrobial Activity Evaluation

**Figure 1:**
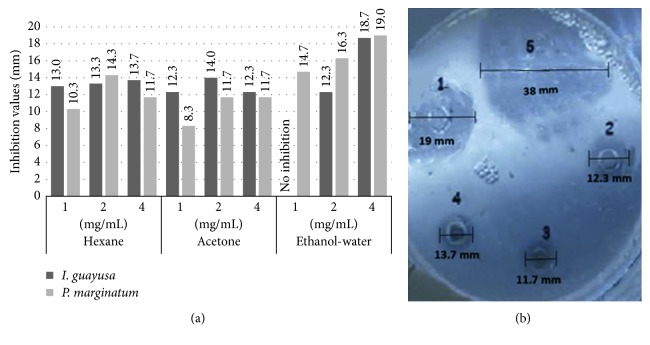
*I. guayusa* Loes and *P. marginatum* Jacq fraction antimicrobial activity against *P. gingivalis* ATCC 33277 at three concentrations. (a) Antimicrobial activities (inhibitory halos mm) from *I. guayusa* Loes and *P. marginatum* Jacq fractions against *P. gingivalis.* (b) 1: *P. marginatum* Jacq 4 mg/mL ethanol : water fraction, 19.0 mm inhibitory halo; 2: *I. guayusa* Loes 4 mg/mL acetone fraction, 12.3 mm inhibitory halo; 3: *P. marginatum* Jacq 4 mg/mL acetone fraction, 11.7 mm inhibitory halo; 4: *I. guayusa* Loes 4 mg/mL hexane fraction, 13.7 mm inhibitory halo; 5: 100 *µ*g/mL ampicillin positive control, 38.0 mm inhibitory halo.

**Figure 2:**
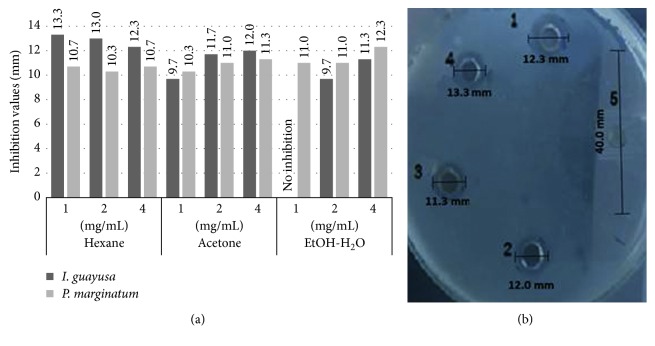
*I. guayusa* Loes and *P. marginatum* Jacq fraction antimicrobial activity against *P. intermedia* ATCC 25611 at three concentrations. (a) Antimicrobial activities (inhibitory halos mm) from *I. guayusa* Loes and *P. marginatum* Jacq fractions against *P. intermedia.* (b) 1: *P. marginatum* Jacq 4 mg/mL ethanol : water fraction, 12.3 mm inhibitory halo; 2: *I. guayusa* Loes 4 mg/mL acetone fraction, 12.0 mm inhibitory halo; 3: *P. marginatum* Jacq; 4 mg/mL acetone fraction, 11.3 mm inhibitory halo; 4: *I. guayusa* Loes 4 mg/mL hexane fraction, 13.3 mm inhibitory halo; 5: 50 IU/mL erythromycin positive control, 40 mm inhibitory halo.

From 1 g *Piper marginatum* Jacq total ethanol extract, hexane, acetone, and ethanol : water fractions were obtained with the following yields: 0.81%, 5.2%, and 55.18%. Likewise, hexane, acetone, and ethanol : water fractions were obtained with yields of 0.23%, 2.52%, and 64.91%, respectively, from 1 g *Ilex guayusa* Loes total ethanol extract. *Ilex guayusa* Loes and *P. marginatum* Jacq total ethanol extract antimicrobial activity against *F. nucleatum* ATCC 25586, *P. gingivalis* ATCC 33277, and *P. intermedia* ATCC 25611 is presented in Table 2. *Ilex guayusa* Loes total ethanol extract against *F. nucleatum* ATCC 25586 did not exert any antimicrobial activity. In contrast, *Ilex guayusa* Loes total extract did have an effect on *P. gingivalis* ATCC 33277 and *P. intermedia* ATCC 25611, with a minimum inhibitory concentration (MIC) of 1 mg/mL. On the other hand, *Piper marginatum* Jacq total ethanol extract exerted antimicrobial activity on all three microorganisms evaluated with a MIC of 1 mg/mL. *Ilex guayusa* Loes inhibitory activity presented halos between 9.3 to 13.7 mm. In contrast, *P. marginatum* Jacq total extracts inhibitory halos were between 12.3 to 30 mm. It is important to highlight the largest inhibitor halo from both plant extracts presented was against *P. gingivalis* ATCC 33277 (Table 2). Likewise, Table 2 shows the antimicrobial activity of the positive and negative controls. Antimicrobial activity from fractions obtained from *Ilex guayusa* Loes and *P. marginatum* Jacq total ethanol extractions against *F. nucleatum* ATCC 25586, *P. gingivalis* ATCC 33277, and *P. intermedia* ATCC 25611 are described in Table 3. None of the negative controls showed antimicrobial activity (Table 3). *Ilex guayusa* Loes obtained fractions presented diverse activities on the three microorganisms studied (Table 3, Part A). *Ilex guayusa* Loes hexane fraction presented a MIC against *F. nucleatum* ATCC 25586 of 4 mg/mL and 1 mg/mL against *P. gingivalis* ATCC 33277 and *P. intermedia* ATCC 25611. Additionally, a 2 mg/mL MIC was observed for acetone fraction against *F. nucleatum* ATCC 25586 and 1 mg/mL against *P. gingivalis* ATCC 33277 and *P. intermedia* ATCC 25611. Moreover, for ethanol : water fraction, a 4 mg/mL MIC against *F. nucleatum* ATCC 25586 and 2 mg/mL against *P. gingivalis* ATCC 33277 and *P. intermedia* ATCC 25611 (Table 3; Figures 1 and 2). In regards to *Piper marginatum* Jacq, not all obtained total ethanol extract fractions presented activity against the three microorganisms studied. Hexane fraction did not present antimicrobial activity against *F. nucleatum* ATCC. However, it did so against *P. gingivalis* ATCC 33277 and *P. intermedia* ATCC 25611 with a MIC of 1 mg/mL. Moreover, acetone fraction presented MIC of 2 mg/mL against *F. nucleatum* ATCC 25586 and 1 mg/mL against *P. gingivalis* ATCC 33277 and *P. intermedia* ATCC 25611. Finally, ethanol : water fraction presented a 1 mg/mL MIC against all three evaluated bacteria (Table 3; Figures 1 and 2).

## 4. Discussion

Identification of plants with pharmacological activity is the main objective of medicinal plant research. Additionally, new molecule discovery presenting antimicrobial activities can be derived from medicinal plants, which can be transformed into possible medications. Furthermore, the medications can be later used as antimicrobial therapeutic agents for infectious diseases prevention and control [15].

Due to its privileged geographical location, Colombian flora is widely known and considered an important source of pharmacological activity products [32]. At present, many substances obtained from plants have been evaluated against pathogenic microorganisms and have demonstrated and/or confirmed antimicrobial activity [33].

It is noteworthy mentioning natural compounds with antimicrobial action are the basis of a line of research dedicated to the discovery of structural and functional components, known as active principles [33]. Research with these compounds is the right path towards efficient and accessible medication development, which can be implemented for treatment of important public health diseases [16–18, 23, 34].

Chronic periodontitis is considered an infectious multibacterial disease, caused principally by obligate anaerobic bacteria [1–3, 35]. These microorganisms interact with tissues and host cells, causing an ample range of cytokine, chemokines, and inflammatory mediator release, which lead to periodontal structure destruction [6–9, 11, 12]. Thus, the need to design measures to evaluate natural products aimed at control or elimination of these microorganisms in the oral cavity; particularly, the impact oral health has on general health and the quality of life of individuals [7–9].

*Piper marginatum* Jacq is a plant belonging to the *Piperaceae* family. In Colombia, it is known as “tooth-healer” or “small cord” [27, 28, 36]. On the other hand, *Ilex guayusa* Loes, known as “guayusa,” is a native plant of the neotropics with natural distribution in Colombia, Ecuador, Peru, Bolivia, and Brazil [29, 37]. In previous studies, different extracts with antimicrobial activity derived from species of the *Piper* genus and *Ilex guayusa* Loes have been evaluated [38–40]. However, up to now, none have addressed *Piper marginatum* Jacq and *Ilex guayusa* Loes extract antimicrobial activity against important microorganisms of periodontal disease. Therefore, the objective of this study was to determine *Piper marginatum* Jacq and *Ilex guayusa* Loes total ethanol extract and fractions antimicrobial activity against periodontal disease microorganisms.

Qualitative phytochemical analysis performed on total ethanol extracts from both plants in this study confirmed appearance of previously reported compounds in the literature [33, 41]. Presence of alkaloid compounds was determined for both plants by means of Dragendorff, Valser, Mayer, and Wagner tests, to which antimicrobial activity is attributed, as it acts on bacterial cell wall and DNA [41]. The Shinoda test on total ethanol extract from both plants detected the presence of flavonoid compounds. According to previous investigations, it has been reported they act on nucleic acid synthesis and prompt bacterial membrane cytoplasmic degradation [41]. For both plants presence of tannins, acting on bacterial adhesins and proteins of the cell wall, with great capacity to bind to extracellular polysaccharides, was established by the ferric chloride test [33]. Additionally, carotenoids and cardiotonic compounds were determined from total extracts obtained from both plants. For its part, coumarin presence was only observed in *Ilex guayusa* Loes total ethanol extract. Antimicrobial properties are credited to this compound, given its capacity to interact with bacterial DNA [41].

Suffredini et al. evaluated various *Piper arboreum* Aubl extract activity against *Staphylococcus aureus*, *Enterococcus faecalis*, *Escherichia coli*, and *Pseudomonas aeruginosa* [38]. *Piper arboreum* Aubl extracts only presented activity against *S. aureus* and *E. faecalis* with a MIC of 60 and 80 *µ*g/mL, respectively. In another study with *Ilex guayusa* Loes and *Piper lineatum* ethanol, methanol and hydroalcohol extracts resulted in antibacterial activity against *S. aureus*, *S. epidermidis*, *Bacillus subtilis*, *E. coli*, and *P. aeruginosa*. Antifungal activity against *Candida albicans* and *Microsporum canis* was also observed [42]. Later, Sánchez et al. [43] determined *Piper marginatum* Jacq essential oil antimicrobial activity against Gram-negative bacteria and *Alternaria solani* Sor fungus [43]. Essential oil at 2.18% demonstrated inhibitory activity against *Xanthomonas albilineans*, *Xanthomonas campestris,* and *A. solani* Sor. However, no activity was observed against *Pseudomonas* [43]. *Piper adumcum* L, *Piper auritum* Kunth, *Piper jericoense* Trel. and Yunck., *Piper obrutum* Trel. and Yunck, and *Piper marginatu*m Jacq antiplasmodial and cytotoxic activity was evaluated by Mesa et al. [39]. From their study, it was concluded moderate antiplasmodial and low cytotoxic activities were observed from extracts obtained from species of the *Piper* genus. On the other hand, *Piper betle* extracts reduced adherence by *Actinomyces* sp, *Streptococcus sanguinis*, and *Streptococcus mitis* to the early plaque in the oral cavity [44]. Another investigation evaluated antibacterial activity in five indigenous plants against human bacterial pathogens, among them, *Piper betle* [15]. *Piper betle* ethanol and methanol extracts presented inhibitory action against *E. coli*, *Klebsiella pneumoniae*, *Salmonella typhimurium*, *S. aureus*, and *Bacillus cereus* [15]. In contrast, Villacis-Chiriboga and collaborators evaluated *Ilex guayusa* Loes aqueous and hydroalcoholic extracts activity against *E. coli* ATCC 25922 and *S. aureus* ATCC 25923, not finding any antibacterial properties [40].

In the present study, outstanding antimicrobial activity was observed from *Piper marginatum* Jacq and *Ilex guayusa* Loes total ethanol extracts and fractions against three evaluated periodontopathogens (*F. nucleatum* ATCC 25586, *P. gingivalis* ATCC 33277, and *P. intermedia* ATCC 25611). *Ilex guayusa* Loes total ethanol extract presented the least activity against all three microorganisms evaluated in comparison with *Piper marginatum* Jacq total ethanol extract; specifically no inhibitory action was observed against *F. nucleatum* ATCC 25586 at the three evaluated concentrations. Nonetheless, it did present activities against *P. gingivalis* ATCC 33277 and *P. intermedia* ATCC 25611. On the contrary, *Piper marginatum* Jacq total ethanol extract did present inhibitory action against all three evaluated microorganisms, particularly with greatest activity against *P. gingivalis* ATCC 33277 (21.7 to 30 mm inhibitory halos). Similarly, *Ilex guayusa* Loes and *Piper marginatum* Jacq hexane, acetone, and ethanol : water fractions presented lower inhibitory activity against *F. nucleatum* ATCC 25586 in comparison with the other two bacteria. Most likely, these diverse susceptibilities obey to intrinsic particularities innate to each anaerobic microorganism evaluated [45]. Furthermore, *Ilex guayusa* Loes acetone fraction and *Piper marginatum* Jacq ethanol : water fraction presented the greatest inhibitory activity against all microorganisms evaluated. These findings demonstrate better recovery of biologically active compounds, as a consequence of charge differences between stationary phase and solvents. In the present study, solvents with different polarities, ethanol : water (high polarity) and acetone (medium polarity), were employed in contrast with amberlite (apolar) in the stationary phase [15].

Performed phytochemical analyses on total ethanol extracts from both plants revealed the presence of various classes of compounds, among them, phenols and flavonoids. Cowan [46] reported phenols, phenolic acid, and quinones are main components in plants with antimicrobial activity. Phenol concentration from total extracts was 57.7 mg gallic acid per gram of *Ilex guayusa* Loes and 45.4 mg gallic acid per gram of *Piper marginatum* Jacq. On the other hand, flavonoid content per gram of *Piper marginatum* Jacq and *Ilex guayusa* was 0.65 mg and 1.71 quercetin. According to studies performed on *Piper marginatum* Jacq by other authors [43, 47, 48], exceptional antimicrobial activity from total ethanol extract and factions from this plant could be attributed to presence of secondary metabolites, such as alkaloids, neolignans, terpenoids, flavones, flavonoids, propenyl phenols, aliphatic amides, and aromatic amides.

Results in this study demonstrate total ethanol extracts and fractions obtained from *Piper marginatum* Jacq and *Ilex guayusa* Loes leaves potential, as a source of various components, with antimicrobial activity against microorganisms of importance in periodontal disease etiology. To this end, more studies to isolate, characterize, and identify active substances in fractions are required. Additionally, antimicrobial activity against an ample gamut of microorganisms important in other oral infections must be determined. In the future, these compounds could be employed in tooth pastes, mouthwashes, and other products of oral hygiene care.

## 5. Conclusions

*Piper marginatum* Jacq and *Ilex guayusa* Loes total ethanol extracts and fractions presented outstanding antimicrobial activity against *P. gingivalis* ATCC 33277, *P. intermedia* ATCC 25611, and *F. nucleatum* ATCC 25586.

## Figures and Tables

**Table 1:** *P. marginatum* Jacq and *Ilex guayusa* Loes total ethanol extract phytochemical analyses.

Secondary metabolite	Test	*Piper marginatum Jacq*	*Ilex guayusa* Loes
Alkaloids	Dragendorff	+	+
	Valser	+	+
	Mayer	+	+
	Wagner	+	+
Cardiotonics	Baljet	+	+
	Molisch	+	+
Carotenoids	Salkowiski	+	+
Coumarins	Ehrlich	−	+
	Fluorescence	−	+
Steroids	Liebermann–burchard	−	−
	Vanille- orthophosphoric acid	−	−
Flavonoids	Shinoda	+	+
	Rosenheim	−	−
	Leucoanthocyanidin	−	−
Sesquiterpenic lactones	Ferric hydroxamate	−	−
Saponins	Froth	−	−
	Rosenthaler	−	−
Tannins	Ferric chloride	+	+
Phenols	Gallic acid	+	+
Flavonoids	Quercetin	+	+

+: positive test result; −: negative test result.

**Table 2:** *Ilex guayusa* Loes and *Piper marginatum* Jacq total ethanol extract antimicrobial activity.

Microorganisms	Concentration (mg/mL)^*∗*^						Controls^*∗*^			
	*Ilex guayusa* Loes			*Piper marginatum* Jacq			Erythromycin (50 IU/mL)	Ampicillin (100 *µ*g/mL)	DMSO	Ethanol
	1	2	4	1	2	4				
*F. nucleatum* ATCC 25586	0.0	0.0	0.0	12.3	13.0	14.3	4.7	4.5	0.0	0.0
*P. gingivalis* ATCC 33277	10.7	13.7	13.3	21.7	27.0	30.0	22.7	38.0	0.0	0.0
*P. intermedia* ATCC 25611	9.3	10.0	11.0	12.3	13.0	15.7	40	14.7	0.0	0.0

Total ethanol extracts were evaluated at 1, 2, and 4 mg/mL on the three microorganisms included in this study. ^∗^Inhibitory halo values in mm, average of three measurements.

**Table 3:** Antimicrobial activity of *Ilex guayusa* Loes (A) and *Piper marginatum* Jacq (B) fractions at 1, 2, and 4 mg/mL with amberlite stationary phase on the three microorganisms evaluated in this study.

Microorganisms	Fraction concentration (mg/mL)									Controls^*∗*^			
	Hexane			Acetone			Ethanol-water			DMSO	Hexane	Acetone	Ethanol-water
	1	2	4	1	2	4	1	2	4				
(A) *Ilex guayusa* Loes^*∗*^													
*F. nucleatum* ATCC 25586	0.0	0.0	12.3	0.0	12.7	13.0	0.0	0.0	10.7	0.0	0.0	0.0	0.0
*P. gingivalis* ATCC 33277	13.0	13.3	13.7	12.3	14.0	12.3	0.0	12.3	18.7	0.0	0.0	0.0	0.0
*P. intermedia* ATCC 25611	13.3	13.0	12.3	9.7	11.7	12.0	0.0	9.7	11.3	0.0	0.0	0.0	0.0

(B) *Piper marginatum* Jacq^*∗*^													
*F. nucleatum* ATCC 25586	0.0	0.0	0.0	0.0	10.7	12.0	10.3	10.7	14.7	0.0	0.0	0.0	0.0
*P. gingivalis* ATCC 33277	10.3	14.3	11.7	8.3	11.7	11.7	14.7	16.3	19.0	0.0	0.0	0.0	0.0
*P. intermedia* ATCC 25611	10.7	10.3	10.7	10.3	11.0	11.3	11.0	11.0	12.3	0.0	0.0	0.0	0.0

^∗^Inhibitory halo values in mm, average of three measurements.

## Data Availability

The data used to support the findings of this study are included within the article and are available from the corresponding author upon request.
